# mRNA Signatures in Peripheral White Blood Cells Predict Reproductive Potential in Beef Heifers at Weaning

**DOI:** 10.3390/genes14020498

**Published:** 2023-02-15

**Authors:** Priyanka Banerjee, Wellison J. S. Diniz, Rachel Hollingsworth, Soren P. Rodning, Paul W. Dyce

**Affiliations:** Department of Animal Sciences, Auburn University, Auburn, AL 36849, USA

**Keywords:** beef heifer, reproductive potential, transcriptome, weaning

## Abstract

Reproductive failure is a major contributor to inefficiency within the cow-calf industry. Particularly problematic is the inability to diagnose heifer reproductive issues prior to pregnancy diagnosis following their first breeding season. Therefore, we hypothesized that gene expression from the peripheral white blood cells at weaning could predict the future reproductive potential of beef heifers. To investigate this, the gene expression was measured using RNA-Seq in Angus–Simmental crossbred heifers sampled at weaning and retrospectively classified as fertile (FH, *n* = 8) or subfertile (SFH, *n* = 7) after pregnancy diagnosis. We identified 92 differentially expressed genes between the groups. Network co-expression analysis identified 14 and 52 hub targets. ENSBTAG00000052659, *OLR1*, *TFF2*, and *NAIP* were exclusive hubs to the FH group, while 42 hubs were exclusive to the SFH group. The differential connectivity between the networks of each group revealed a gain in connectivity due to the rewiring of major regulators in the SFH group. The exclusive hub targets from FH were over-represented for the CXCR chemokine receptor pathway and inflammasome complex, while for the SFH, they were over-represented for immune response and cytokine production pathways. These multiple interactions revealed novel targets and pathways predicting reproductive potential at an early stage of heifer development.

## 1. Introduction

Reproductive efficiency has declined over time, negatively impacting beef cattle production systems and the livestock industry [1]. Reproductive failure contributes as one of the main reasons for culling breeding-age female heifers, which reduces a herd’s longevity and affects producer profits [2]. Significant efforts are placed on the morphological assessment and selection of heifers before the breeding season. Body condition score, reproductive tract score, and pelvic measurements are the main traits used by producers to select heifers with a high reproductive potential [3,4,5,6]. However, a persistent population of heifers deemed reproductively mature fails to conceive. Improving heifer fertility utilizing genetic selection is becoming increasingly important as declining fertility cannot be arrested by only improving management [7]. Therefore, there is an increased demand to develop a system that could discriminate beef heifers with varying reproductive potentials as early and accurately as possible.

To fulfill those demands, genome-wide expression profiling has been used in functional genomics to unravel the coordinated functioning of cells and tissues regulating biological mechanisms at the transcriptional level [8]. Transcriptome sequencing has revolutionized the field and provided opportunities to profile gene expression to identify the molecular mechanisms affecting fertility. In bovines, a large number of transcriptomic studies from embryo [9,10], endometrium [11,12,13,14], uterine tissue [15], and peripheral white blood cells (PWBCs) [16,17,18] have reported candidate genes associated with differing pregnancy outcomes. Binelli et al. identified *FRAS1*, *DIO2*, *ADAM12*, *KIAA1324L*, *LRRC4C*, *TMEM61*, *AK5*, and *PIPOX* genes differentially expressed in uterine endometrial biopsies collected during early pregnancy to differentiate between non-pregnant and pregnant cows [15]. Similarly, Dickinson et al. reported genes, such as *ALAS2*, *CNKSR2*, *LOC522763*, *SAXO2*, *TAC3*, and *TFF2,* differentially expressed in PWBCs between pregnant and non-pregnant beef heifers measured at the time of artificial insemination [17]. The studies mentioned above are informative and provide potential candidates for identifying heifers with high reproductive potential; however, there is a gap in knowledge in most studies. First, most of the studies are conducted during early pregnancy or at the time of artificial insemination (AI). By this time point, it may be too late for the producer who has invested time and money in developing replacement heifers (young fertile cows). Second, all the studies identified differentially expressed genes between the animals of varying reproductive potential. However, due to regulatory mechanisms, changes in gene expression patterns may not always have significant consequences on a biological pathway. Furthermore, the differential gene expression approach disregards the multiple gene interactions affecting the regulation [19]. 

One of the ways to accomplish the first limitation is to identify candidates at an early stage of heifer development, such as “at weaning” when the replacement heifers are traditionally selected. One of the most reliable, non-invasive, and predictable methods to identify candidate genes is from blood. Studies have reported that RNA in the blood reflects the health, phenotype, and developmental status of organs in mammals [20,21]. Interestingly, infertility issues and pregnancy outcomes can be predicted through blood biomarkers in humans [22,23] and cattle [16,17]. To overcome the second limitation, gene co-expression networks provide a framework to identify genes with coordinated expression that underlie regulatory processes [24]. This approach focuses on hub targets (highly connected genes) that translate predictions to testable hypotheses [25]. Additionally, differential network analysis identifies gene interconnections between groups, indicates differences in biological mechanisms, and identifies networks rewired under different conditions [26,27,28]. 

Thus, our main objective was to investigate the molecular basis of beef heifer fertility, predict reproductive potential at weaning, and shed light on multiple gene interactions regulating biological mechanisms involved with gene expression and reproductive outcome. We hypothesized that genes are differentially co-expressed and gene networks are rewired in beef heifers at an early developmental stage, contributing to varying reproductive outcomes at maturity. 

## 2. Materials and Methods

### 2.1. Animal Handling and Phenotype Collection

The Institutional Animal Care and Use Committee (IACUC) at Auburn University and the guide for the Care and Use of Laboratory Animals (IACUC protocol numbers 2015-2786 and 2019-3591) approved all procedures involving animals. 

Crossbred heifers (Angus-Simmental) used in this study were developed as replacement heifers at the Alabama Research and Extension Centers (Auburn University). At the time of weaning (~238 days after birth), blood samples (10 mL) from 75 heifers were collected into vacutainers containing EDTA (Becton, Dickinson and Company, Franklin Lakes, NJ, USA) from the jugular vein. The blood was processed to extract PWBCs as described elsewhere [17]. In brief, the vacutainer tubes were centrifuged at 1500× *g* for 10 min at 4 °C. The buffy coat was aspirated and added to a fresh tube with 15 mL red blood cell lysis buffer (0.15 M ammonium chloride, 10 mM potassium bicarbonate, 0.1 mM EDTA, Cold Spring Harbor Protocols) and incubated for 10 min at room temperature. The solution was centrifuged at 500× *g* for 5 min at 4 °C to pellet the PWBCs. The supernatant was discarded, and the pellet was resuspended in 1.5 mL phosphate buffer saline + 2% of fetal bovine serum. The tube was centrifuged at 500× *g* for 5 min at 4 °C. The supernatant was discarded and PWBCs were stored at −80 °C until further processing. Phenotypic data, such as weaning weight and age, were recorded for each heifer. 

**Figure 1:**
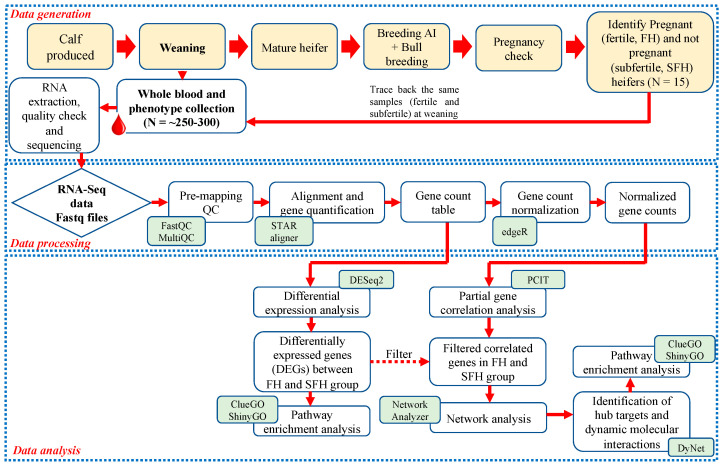
Experimental design and the bioinformatics workflow of the study.

During the breeding season, 72 heifers were selected based on ideal body condition scores (5–6) and reproductive tract scores (≥4). All heifers underwent the same estrus synchronization, and fixed-time AI protocol (7-Day CO-Synch + CIDR) [17]. The heifers received 100 μg GnRH (Cystorelin; Merial Inc., Duluth, GA, USA) via intramuscular injection and an intravaginal insert of a controlled internal drug release (CIDR) device containing 1.38 g of progesterone (Eazi-Breed CIDR; Zoetis Inc., Kalamazoo, MI, USA). CIDRs were removed following 7 days and an intramuscular injection of 25 mg of dinoprost tromethamine (Lutylase, Zoetis Inc., Kalamazoo, MI, USA) was administered at the same time. After a second intramuscular injection of GnRH (100 μg), heifers were artificially inseminated with a single straw of semen from Angus sires 54 ± 2 h following CIDR removal. Fourteen days following the fixed-time AI, all heifers were exposed to fertile bulls for a 60-day natural breeding season. Bulls used at the facility were proven breeders and passed a standard breeding soundness examination (BSE). This 60-day natural breeding season was to allow sufficient opportunity for heifers to conceive. Heifers that did not conceive after fixed-time AI followed by a 60-day natural breeding season with bulls that satisfactorily passed a BSE were considered subfertile for the purpose of this study. Therefore, depending on the presence or absence of conceptus at 75 days following AI, heifers were classified as fertile for those that were pregnant to AI (*n = 35*, FH), pregnant to natural breeding (*n = 26,* P-NB), or non-pregnant (*n = 11*, SFH). Heifers that were pregnant to AI (FH, *n* = 8) or non-pregnant (SFH, *n* = 7) and that had comparable birth age, weaning age, and body weight were considered for the study. The experimental design and the bioinformatics workflow of the study are shown in Figure 1.

### 2.2. RNA Extraction, Library Preparation, and Sequencing

According to the heifer classification described above, total RNA was extracted from the PWBCs of 15 samples (FH and SFH) collected at the time of weaning. The total RNA was extracted using Trizol reagent (Invitrogen, Carlsbad, CA, USA) following standard procedures. RNA purification and DNase digestion were performed using an RNA clean and concentrator kit (Zymo Research, Irvine, CA, USA). The quality and RNA integrity of total RNA was assessed using Agilent Bioanalyzer, following the Agilent RNA 6000 Nano kit (Agilent, Santa Clara, CA, USA) instructions. The samples with average RIN values > 6.0 were further subjected to sequencing. Library preparation and sequencing were performed on the Nova-Seq platform at Discovery Life Sciences (Hudson Alpha Institute of Biotechnology, Huntsville, AL, USA). Paired-end 100 bp reads were generated for each sample.

### 2.3. Data Processing and Differential Expression

The quality of raw sequencing data was checked using FastQC v0.11.9 [29] and MultiQC v1.12 [30]. The quality of the reads was evaluated based on parameters such as average read length, adapter content, per sequence GC content, and sequence quality scores. The reads were mapped using STAR aligner v2.7.5 to Ensemble’s ARS UCD1.2 *Bos taurus* genome reference (https://useast.ensembl.org/Bos_taurus/Info/Index, accessed on 14 August 2022) [31]. The read quantification was performed using the STAR with the *-quantMode* gene counts flag to obtain the raw counts per gene. Read counts were transformed to counts per million (CPM) using edgeR v3.28.1 [32]. Post-mapping quality control was performed using MultiQC v1.12. Genes with a low count (expression) with CPM < 1 in 50% of the samples (*n* = 8) were filtered out. The differentially expressed genes (DEGs) were identified using DESeq2 v1.26.0 [33]. The contribution of the phenotypes such as weaning age, weight, birth weight, and RIN values to the total gene expression was checked with ANOVA and principal component analysis prior to differential expression analysis. The pregnancy status (fertile or subfertile) was considered for the design model used on DESeq2. The DEGs with *padj* ≤ 0.05 and absolute (log2 fold change) ≥0.5 were considered significant. The SFH group was considered as the reference and the DEGs up or downregulated were classified based on the log2 fold change direction. To visualize the DEGs, we created a volcano plot using the EnhancedVolcano v1.4.0 R-package [34]. 

### 2.4. Co-Expression Profile, DEGs Filtering, and Gene Networks

To identify the gene-gene interactions, the partial correlation and information theory (PCIT) algorithm was used. PCIT considers concepts of partial correlation and mutual information and compares all possible triplets of genes to report the significant gene pairs that are tightly correlated over a small expression range [35]. The raw read counts were normalized with CPM using edgeR v3.28.1 and used as the input for PCIT analysis. Separate gene networks were constructed for the FH and SFH groups. For each group, gene correlations with *p*-value ≤ 0.05 and absolute r ≥ 0.95 were considered significant. Additionally, both networks were filtered to identify the gene pairs co-expressed with the DEGs. The FH and SFH networks were analyzed using the Network Analyzer tool in Cytoscape v3.8.2 [36] to identify “hub targets”. The hub targets were identified by considering the mean + 2 SD (standard deviation) of the degree measure in the network. The visualization of nodes (genes) and edges (Interactions) from the FH and SFH groups was performed using the DyNet plugin in Cytoscape v3.8.2. 

To identify the differentially connected genes, the network connectivity measures from each group were standardized by taking the ratio of gene connectivity and maximum connectivity [37]. The differential connectivity (DK) was calculated as $DK_{i}=K_{SFH\left(i\right)}-K_{FH\left(i\right)}$. The DK values were transformed to z-scores and ± 1.96 SD (*p*-value ≤ 0.05) was considered significant. The networks were visualized using Cytoscape v3.8.2.

### 2.5. Pathway Analysis

Pathway over-representation analyses based on the DEGs and hub targets exclusive for FH and SFH groups were carried out using ClueGO v2.5.4 (Cytoscape plug-in) [38] and ShinyGO v0.76 [39]. In ClueGO, the *Bos taurus* annotation was used as the background. Additionally, redundant terms were clustered based on the kappa score (kappa = 0.4). The *p*-values were corrected with the Bonferroni method, and *padj* ≤ 0.05 was considered significant. The parameters to retrieve pathways from ShinyGO included *Bos taurus* as the reference and the FDR cutoff ≤0.05. 

### 2.6. mRNA Expression of Top Targets at Weaning with RT-qPCR

The significant up/downregulated genes, also identified as hub targets, were validated in a separate set of 10 PWBC RNA samples (FH = 5, SFH = 5) collected at weaning from heifers housed at the Alabama Research and Extension Center (Auburn University). The total RNA was extracted using the Trizol protocol, as mentioned in Section 2.2. The RNA concentration was checked using a Qubit RNA broad-range assay kit (Life Technologies, Thermo Fisher Scientific Inc., MA, USA) on a Qubit Fluorometer v3.0. The total RNA was reverse transcribed using qScript cDNA Supermix (Quanta Biosciences Inc., Beverly, MA, USA). The complementary DNA (cDNA) was diluted 1:5 (v:v). The final reaction constituted of 2 μL diluted cDNA, 100 nM of each primer, and PerfeCTa SYBR green supermix (Quanta Biosciences Inc., Beverly, MA, USA) to a total reaction volume of 10 μL. The primer sequences used for RT-qPCR are given in Table S1. The reactions were assayed in Roche Light Cycler 480 (Roche Diagnostics, IN, USA). The fold change in the mRNA expression was calculated using the ΔΔCt method [40] by normalizing the cycle threshold value of the targeted gene of interest to the housekeeping gene *GAPDH* included in each plate. 

### 2.7. Statistical Analysis

The statistical analysis for RT-qPCR and the graphs were constructed using GraphPad Prism v6.01 (GraphPad, SanDiego, CA, USA), and the results are reported as mean ± SD. The Mann–Whitney test (non-parametric) was used for the statistical analysis, and a *p*-value ≤ 0.05 was considered statistically significant. 

## 3. Results

### 3.1. Transcriptome Profiling from PWBCs and Differential Expression Analysis

We used RNA-Seq expression profiles from PWBCs to ascertain the potential biological pathways underlying differing pregnancy outcomes between FH (*n* = 8) and SFH (*n* = 7) groups. The sequencing from all the samples yielded an average of 28.6 million reads per sample, of which 93.5% were uniquely mapped to the *Bos taurus* genome (Table S2). After filtering non-expressed or lowly expressed genes, we identified 12,270 genes out of 27,607 genes in 15 samples for further analysis. 

**Figure 2:**
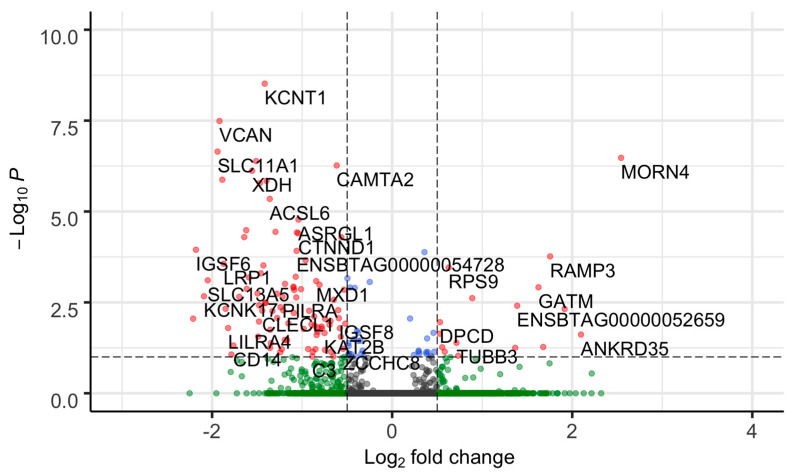
Volcano plot of differentially expressed genes between FH and SFH groups. Each dot corresponds to a gene. The difference in gene expression between the FH and SFH groups is shown as the log2 fold change (x-axis). The negative log (base 10) of the *p*-value is shown on the y-axis. Gene significance is color coded as grey (non-significant genes that did not cross the threshold of *padj* value or fold-change); green (genes with absolute (log2 fold change ≥ 0.5)); blue (genes with a significant *p*-value); and red (92 DEGs with *p*-value ≤ 0.05 and absolute (log2 fold change ≥ 0.5)). The genes were classified as up- or downregulated based on the sign of the log2 fold change. Negative values (0 to −3 of log2 fold change) were downregulated, and positive values (0 to 4 of log2 fold change) were upregulated.

We identified 92 DEGs between FH and SFH groups out of 12,270 genes ((*padj* ≤ 0.05 and absolute (log2 fold change ≥0.5)) (Figure 2, Table S3). The 92 DEGs included 88 protein-coding genes and two each of miscellaneous and long non-coding RNA (Table S3). The top five significantly upregulated genes in the SFH group with the highest log2 foldchange difference included *GATM, MORN4*, *ANKRD35*, *TFF2*, and *RAMP3,* while the downregulated genes included *CLEC4D*, *IGSF6*, *KCNK17*, *SLC13A5*, and *SLC11A1*.

### 3.2. PCIT and Network Analysis

To identify the co-expressed genes within the FH and SFH groups, we constructed two networks using the PCIT algorithm. The correlation of 12,270 resulted in 3,694,087 significantly correlated pairs in the FH group, whereas there were 3,086,024 correlations for the SFH group. To retrieve biologically meaningful information and reduce data dimensionality, we filtered the genes with absolute correlations greater than 0.95 and co-expressed with DEGs (Table S3). After filtering, we retrieved 2024 and 8636 significantly correlated gene pairs for FH and SFH groups, respectively (Table S4). To identify specific network connections and gene connectivity (degree) in each group (FH and SFH), we analyzed each network separately using Cytoscape v3.8.2. Genes with the highest degree (highly connected) are likely to regulate important biological functions and are called hub targets. We identified 14 and 52 hubs in the FH and SFH groups, respectively (Table S5). The top hub targets for the FH network included *ENSBTAG00000039132*, *ENSBTAG00000051464*, *PHF8*, *MXD1*, *ENSBTAG00000052659*, *OLR1*, *MTMR12*, and *TFF2.* Among them, *ENSBTAG00000039132* had the greatest number of connections (co-expressed with 320 genes) in the FH group. The top hub targets for the SFH network included *PHF8*, *KAT2B*, *MTMR12*, *CAMTA2*, *ENSBTAG00000054728*, *IL1R1,* and *ENSTBAG00000039132. PHF8* had the greatest number of connections (558 genes) in the SFH group. After overlapping the 14 and 52 hub targets from both the groups, four hubs—*ENSBTAG00000052659*, *OLR1*, *TFF2*, and *NAIP* were exclusive to FH, while 42 hubs were exclusive to the SFH group, and 10 hubs were shared between the FH and SFH groups (Table S5). 

**Figure 3:**
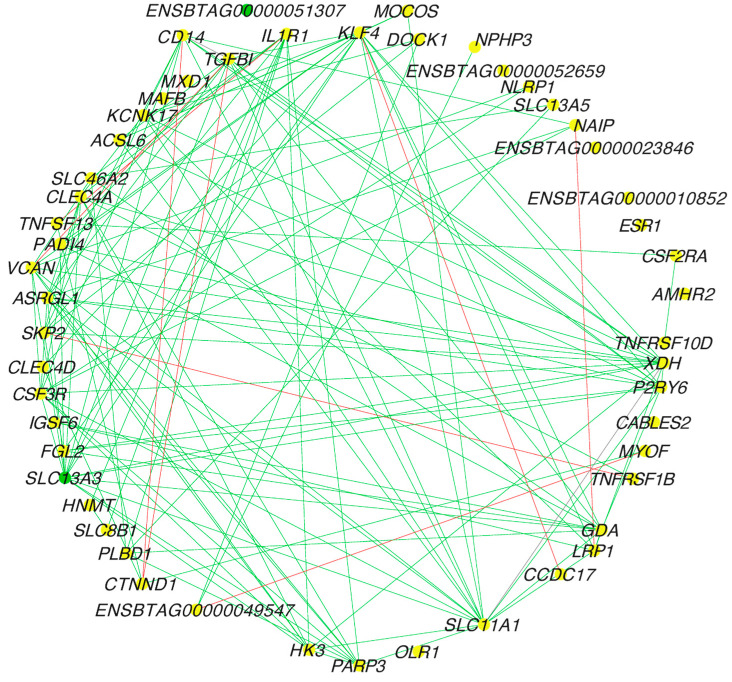
Central reference network of co-expressed genes between FH and SFH groups. The network was constructed using DyNet and visualized on Cytoscape. The network comprises 4190 nodes (genes) and 10,550 edges (interactions). For better visualization, the network was filtered with hub targets based on the degree of connections identified. The unique nodes are green (SFH group) and yellow (shared between FH and SFH groups). The red edges represent the interactions in the FH group, while the green edges represent the interactions in the SFH group.

The central reference network for FH and SFH groups (Figure 3) was constructed using DyNet from 4190 nodes (genes) and 10,550 edges (interactions) (Table S6). Next, we calculated the differential connectivity between the groups. To this end, we calculated the frequency of gene degrees of 4190 genes. The frequency was then transformed into a z-score. Thus, we identified 52 differentially connected genes, out of which 47 genes gained connectivity in the SFH group (*p*-value ≤ 0.05). The top five DEGs that gained connectivity in the SFH group were *VCAN*, *SLC11A1*, *CAMTA2*, *XDH*, and *GDA,* while *TFF2*, *OLR1*, *ENSTBATG00000052659*, *ENSBTAG00000039132,* and *ENSBTAG00000051464* gained connectivity in the FH group.

### 3.3. Functional Over-Representation Analysis

**Figure 4:**
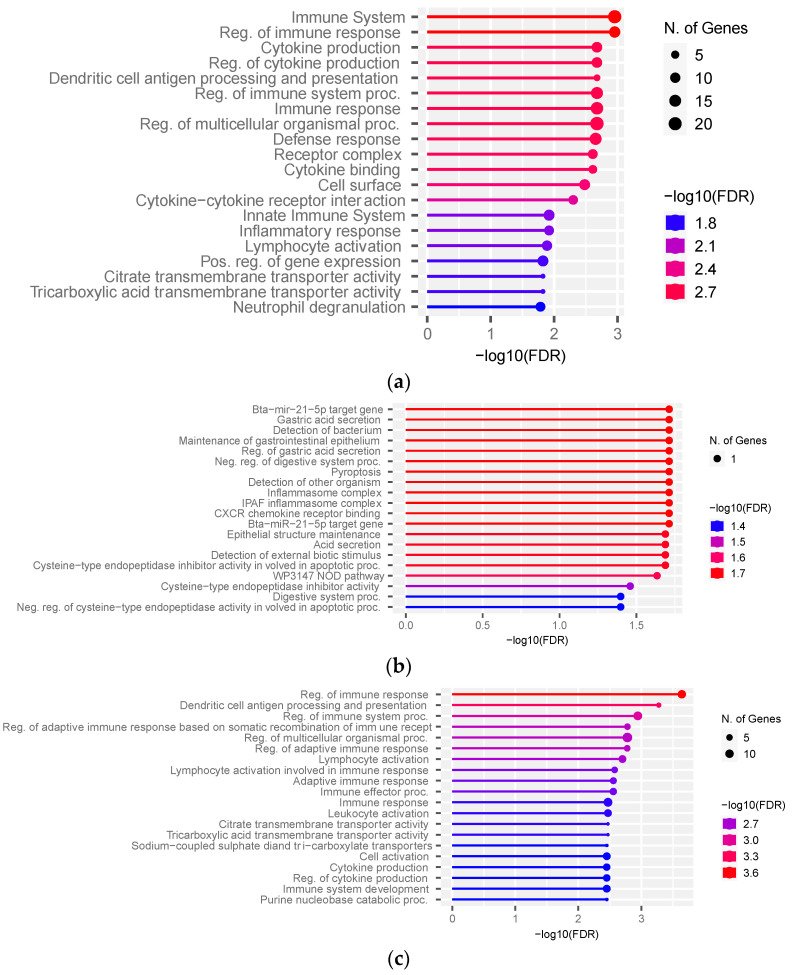
Pathway over-representation analysis of differentially expressed genes and co-expression network hubs between the FH and SFH groups. Pathways are over-represented by (**a**) differentially expressed genes, (**b**) hub targets exclusive to the FH group, and (**c**) hub targets exclusive to the SFH group.

To translate the list of DEGs into meaningful biological data, we performed pathway enrichment analysis. The top pathways identified with ShinyGO v0.76 were the immune system, cytokine production, and defense response (FDR ≤ 0.01) (Figure 4a, Table S7A). The over-represented biological processes and KEGG pathways affected by DEGs identified from ClueGO v2.5.4 pointed to cytokine binding, hematopoietic cell lineage, positive regulation of the reactive oxygen species metabolic process, and the negative regulation of cysteine-type endopeptidase activity involved in the apoptotic process. The exclusive hub targets in the FH group using ShinyGO v0.76 were enriched for inflammasome complex, CXCR chemokine binding receptor, and epithelial structure maintenance (Figure 4b, Table S7B). The hub targets exclusive to the SFH group were enriched for the regulation of immune response, citrate and tricarboxylic acid transmembrane transporter activity, cell activation, and cytokine production (Figure 4c, Table S7C). The 10 shared hub targets did not over-represent significant pathways. Since one gene was over-represented in each pathway identified in the FH group (Figure 4b, Table S7B), we performed the pathway analysis with all the genes co-expressed with four exclusive hub targets from the FH group. No significant pathways (FDR < 0.05) were identified from genes co-expressed with *ENSBTAG00000052659*. The genes co-expressed with *OLR1* in the FH group were enriched for intracellular protein-coding complex, transferase complex, and nuclear body (FDR < 0.05) (Table S8A), while those co-expressed with *TFF2* were enriched for the apoptotic process, interleukin production, and cytokine-mediated signaling pathways (Table S8B). The genes co-expressed with *NAIP* were enriched for the apoptotic process, the cellular response to amyloid beta, and cell proliferation (Table S8C). 

### 3.4. mRNA Expression of the Identified DEGs and Hub Targets

**Figure 5:**
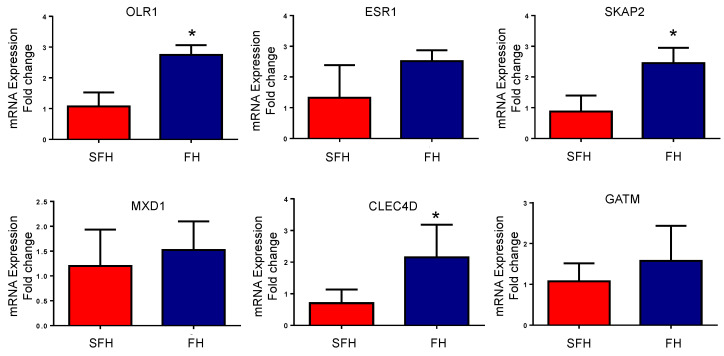
mRNA expression of differentially expressed genes between the fertile heifers (FH) and subfertile heifers (SFH) group based on RT-qPCR. The fold change was evaluated in both groups (FH and SFH). Data are represented as mean ± SD in each group. * *p*-value < 0.05.

From the DEGs and hub target results, six genes were selected to quantify their expression with RT-qPCR. Consistent with the results mentioned above, *CLEC4D*, *SKAP2*, and *OLR1* were significantly downregulated (*p* ≤ 0.05). *ESR1* and *MXD1* were identified with low expression levels on the SFH heifers. The difference in the levels was not significant between the groups (*p*-value ≥ 0.05); however, the trend is the same as identified by the RNA-Seq approach (Figure 5). 

## 4. Discussion

Reproductive performance is critical for the success of beef cattle production. Heifers with high reproductive potential improve a herd’s performance and benefit producers. Many events are involved in optimizing reproductive efficiency, including heifer puberty onset occurring before the breeding season, early conception, calving early in the subsequent calving season, unassisted calving, and calf survival [41]. Any interruption in the preceding cycle contributes to reproductive losses [41]. Phenotypic traits, including body condition score, reproductive tract score, and pelvic measurements, are used by producers to evaluate the reproductive potential of beef heifers and minimize the loss in rearing less productive animals [4,42]. Although these tools are helpful for the early management of the herd, some heifers categorized as reproductively mature fail to conceive after the breeding season. Furthermore, many of these metrics require the heifer to be close to breeding age, limiting the usefulness of the information. Therefore, understanding the genetic basis for reproductive potential at an early stage of development will provide opportunities to efficiently select beef heifers and benefit the cow-calf production system. 

As a step forward, we employed an RNA-Seq framework to measure the expression of the genes from the PWBCs of beef heifers at weaning. We identified 92 DEGs between the FH and SFH groups. To better understand the gene–gene co-expression pattern in both FH and SFH groups, we prioritized candidate genes from differential gene expression analysis to identify specific co-expression differences between the two groups. Then, we used DK analyses to identify hub targets. Apart from identifying the hubs, the DK analysis also identified the differential connectivity of genes in each group. Interestingly, the gene network from the SFH group showed a gain in connectivity, suggesting the rewiring of major regulators that modulate the gene expression of the targets, potentially resulting in failed pregnancy outcomes. Furthermore, we identified biologically meaningful pathways over-represented by the differentially expressed genes and the hub targets in each group.

Among the 92 DEGs and hub targets identified in each group, we identified two genes, *OLR1* (*oxidized low-density lipoprotein receptor 1*) and *LRP1* (*low-density lipoprotein (LDL) receptor-related protein-1*), that were significantly downregulated in the SFH group. Furthermore, *OLR1* was significantly downregulated in mRNA expression levels measured by RT-qPCR in the current study. Both of the genes were over-represented underlying the receptor complex pathways. *OLR1* was significantly more connected in the FH group and a hub target, while *LRP1* was an exclusive hub target with a connectivity gain in the SFH group. This agrees with a study in humans where *OLR1* was downregulated in the blood mononuclear cells of non-pregnant women [43]. Lower expression of *OLR1* during the pre-conceptional period may attribute to a physiological increase in oxidative stress during early pregnancy [43]. Increased oxidative stress has a negative role in pregnancy. It may cause an improper endometrial or immunological preparation for the pregnancy or an adverse environment for the continuation of pregnancy [43]. On the other hand, in pregnant women, the balanced expression of *OLR1* leads to the normal regulation of oxidized low-density lipoprotein, thus leading to normal placentation during the first trimester of pregnancy [43]. In a separate study with ewes, a polymorphism in the *OLR1* gene was associated with reduced fertility traits [44]. Similarly, *LRP1* is highly expressed in the human placenta and is suggested to play a role in placental lipid transport [45]. Attributing to all the reasons mentioned above, the downregulation of *OLR1* and *LRP1* genes and low reproductive potential in the SFH group could be well hypothesized and needs to be further explored in bovines.

Among the hub targets identified in the SFH group, we identified *ESR1* (estrogen receptor 1) as a hub target and over-represented in immune response pathways. *ESR1* transduces the cellular response to estrogen; defects of *ESR1* were reported to be closely related to ovarian defects, including the premature depletion of ovarian follicles or arrested folliculogenesis in humans [46]. Furthermore, estrogen receptor signaling is required for modulating and maintaining pregnancy. Genetic variants in *ESR1* were associated with recurrent pregnancy loss in women [47]. Along with *ESR1*, we identified *SKAP2*, *MXD1*, and *CLEC4D* as downregulated in the SFH group. *ESR1* and *MXD1* showed a low expression level, while *SKAP2* and *CLEC4D* were significantly downregulated (*p* ≤ 0.05) in the mRNA expression level measured by RT-qPCR in our study. *SKAP2* was downregulated in the villous tissues of patients with failed pregnancies [48], while *MXD1* was associated with early and late gestation in patients with severe preeclampsia [49]. Contrastingly, *GATM* was identified as upregulated in the SFH group in our study, but no significant difference was observed in the mRNA expression level measured by RT-qPCR (*p* ≥ 0.05). The elevated mRNA expression of *GATM* was identified in the placenta of patients with preeclampsia [50].

The *TFF2* (*trefoil factor 2*) gene was upregulated in the SFH group. Interestingly, the gene lost connectivity in the SFH group compared to the FH group and was a hub target in the FH group. In the FH group, the gene is over-represented in the CXCR chemokine receptor pathway. The chemokine receptors control ovarian folliculogenesis and fertility in mice [51]. The CXCRs have a potential role in follicular growth during adult life, potentially affecting the reproductive lifespan [51]. Interestingly, in cattle, *TFF2* was upregulated in the blood from SFH beef heifers collected at the time of AI [17,18]. This finding suggests a role of *TFF2* in fertility; its identification in heifers of varying reproductive potential at weaning and later exhibited at AI makes it a potential candidate to discern the reproductive potential at an early developmental stage.

We identified *NAIP* (*NLR family apoptosis inhibitory protein*) as a hub target and upregulated in the FH group. *NAIP* is involved with immune and defense responses, the inflammasome complex, the inhibition of apoptotic activity, and the NOD pathway. *NAIP* is strongly associated with both innate immunity and inflammation [52] and participates in the formation of a signaling platform called NLRC4 inflammasome [53]. During bacterial infection, bacterial proteins in the cytoplasm are detected by NAIPs to activate the NLRC4 inflammasome, which results in the processing and activation of precursors of IL-1β and IL-18 cytokines for extracellular secretion [53]. Coordinated communication between interferons and cytokine signaling pathways promotes a healthy pregnancy [54]. The inactivation or downregulation of *NAIP* and low immunity against pathogenic infections may potentially negatively affect fertility in the SFH group. 

In addition to the immune response genes, *NLRP1* was identified as a hub target in the SFH group. *NLRP1*, other genes of the TNF superfamily (*TNFSF13* and *TNFSF1B*), *MAFB*, *IL1R1*, *PARP3*, *FGL2*, the solute carrier family (*SLC46A2* and *SLC11A1*), genes from the C-type lectin-like domain superfamily (*CLEC4A* and *CLEC4D*), *CSF3R, ESR1*, and *KLF4* were identified as exclusive hub targets and over-represented for immune response pathways in the SFH group. Interestingly, all the genes were downregulated and significantly more connected in the SFH group network. *SLC11A1* plays a role in blocking productive placental infections by intracellular pathogens [55]. Likewise, *KLF4* upregulates a protein PSG-5, known for maintaining pregnancy and protecting the fetus from attack by the maternal immune system [56]. Although host defense depends on inflammation, an uncontrolled inflammatory response may negatively affect fertility [57]. *MAFB*, a transcription factor, is downregulated by lipopolysaccharide (LPS) [58]. LPS is associated with adverse developmental outcomes and impairs fetal growth [59]. A possible explanation for some of the heifers not becoming pregnant could be attributed to the downregulation of the *MAFB* gene in the SFH group. 

*CTNND1* (*Catenin delta 1*) was identified as a hub target and downregulated in the SFH group. *CTNND1* encodes a protein p120-catenin with a broad range of biological roles, including the maintenance of cell–cell junctions, the regulation of the epithelial–mesenchymal transition, and transcriptional signaling [60,61]. The complete deletion of *CTNND1* in mice decreases the microvascular density, reduces pericyte coverage, and disorganizes vascular networks in both embryonic and extraembryonic tissues, eventually leading to prenatal lethality [61]. Although no over-represented pathway included the *CTNND1* gene, its role in the SFH group seems significant. 

Altogether, we identified genes and their interactions that underlie pathways playing a role in the reproductive outcome. Although some genes were previously reported in the literature, we also found novel candidates that need to be validated in a larger cohort. Furthermore, the genes are differentially expressed between the FH and SFH groups at weaning. The validation of these genes at a different time point, such as at AI, on heifers with varying reproductive outcomes, would help establish a framework for reproductive potential prediction at an earlier time point and be used to improve reproductive efficiency.

## 5. Conclusions

The findings reported here indicate significant differences in the PWBC transcriptome profile associated with fertile and subfertile groups of beef heifers at weaning. The originality of this research lies in the multiple approaches used to determine gene interactions at an early stage of development, such as at weaning. The expression of genes, identifying correlated genes, and the underlying pathways identified in this study provide potential targets for the future prediction of reproductive outcomes. A detailed understanding of the underlying biological mechanisms of the top targets and a follow-up study from weaning to AI could validate the potential candidates identified in this study. 

## Data Availability

All the datasets are publicly available on the GEO database (GEO accession ID: GSE221903).

## Supplementary Materials

The following supporting information can be downloaded at: https://www.mdpi.com/article/10.3390/genes14020498/s1. Table S1: Primer sequence for RT-qPCR analysis; Table S2: RNA sequencing summary and mapping statistics; Table S3: Differentially expressed genes between the FH and SFH groups (padj < 0.05 and |log2 fc| > 0.5); Table S4: The genes correlated with 92 differentially expressed genes identified with the PCIT approach, with correlation >0.95; Table S5: Network analysis of genes co-expressed with differentially expressed genes in FH and SFH groups; Table S6: Central reference network including both FH and SFH groups; Table S7A: Pathway over-representation analysis of the differentially expressed genes; Table S7B: Pathway over-representation by hub targets exclusive to FH group with FDR < 0.05; Table S7C: Pathway over-representation by hub-targets exclusive to SFH group with FDR < 0.05; Table S8A: Pathways over-represented with the genes co-expressed with ENSBTAG00000004547 (OLR1) in the FH group, FDR < 0.05; Table S8B: Pathways over-represented with the genes co-expressed with ENSBTAG00000030814 (TFF2) in the FH group, FDR < 0.05; Table S8C: Pathways over-represented with the genes co-expressed with ENSBTAG00000003326 (NAIP) in the FH group, FDR < 0.05.
